# Effect of green fuel and green lubricant with metallic nanoparticles on emissions of HC, CO, NO_*x*_, and smoke for a compression ignition engine

**DOI:** 10.1007/s11356-023-28645-z

**Published:** 2023-07-21

**Authors:** Augustine B. V. Barboza, Pijakala Dinesha, Marc A. Rosen

**Affiliations:** 1 Department of Mechanical and Industrial Engineering, Manipal Institute of Technology, Manipal Academy of Higher Education, Manipal, 576104 India; 2 Faculty of Engineering and Applied Science, University of Ontario Institute of Technology, 2000 Simcoe Street North, Oshawa, ON L1G 0C5 Canada

**Keywords:** Biolubricant, Clean energy, Engine lubrication, Green fuel, Tailpipe emissions

## Abstract

The United Nations Sustainable Development Goals (SDGs) are imperative from the point of view of protecting the environment by employing sustainable options. Considerable research has been carried out in the transportation sector to meet this objective. Here, the influence is assessed of epoxidised gingelly oil methyl ester biolubricant with alumina (Al_2_O_3_) nanoparticles on the performance and emissions of a single cylinder 0.66-L capacity direct injection compression ignition engine driven by gingelly B20 biodiesel. Engine tests are carried out with gingelly B20 biodiesel as a fuel, and gingelly methyl ester (B100), epoxidised gingelly methyl ester (B100E), and epoxidised gingelly methyl ester (B100E) mixed with 0.5%, 1.0%, and 1.5% w/w alumina (Al_2_O_3_) nanoparticles as the lubricant combinations. The results are compared with baseline B20 biodiesel fuel-mineral lubricant operation. The findings indicate that brake thermal efficiency increases by 8.64% for epoxidised gingelly methyl ester (B100E) with 1.0% w/w alumina (Al_2_O_3_) nanoparticle biolubricant in comparison to baseline operation. Considerable reductions in emissions are detected; specifically, reductions of 52.4%, 22.0%, 20.0%, and 34.9%, respectively, are observed for CO, NO_*x*_, and HC concentrations and smoke opacity for the abovementioned combination as compared to baseline operation. The present work suggests that further research is merited on green fuel-green lubricant combinations. The findings of this study address the United Nations Sustainable Development Goals (SDGs) 7 and 13.

## Introduction

Of late the utilisation of biofuels for internal combustion (IC) engines is gaining momentum for environmental and performance reasons. Several studies have reported superior engine performance coupled with reduced tailpipe emissions whilst using biofuel either in neat or blended form, with or without additives (Alruqi et al. 2023; Anantha Padmanabha and Mohanty 2023; Anupong et al. 2023; Elkelawy et al. 2022; Jain et al. 2023; Mohan et al. 2022; Said et al. 2023; Shanmuganathan et al. 2023; Suhel et al. 2023; Wu et al. 2023). Experimentation in most of the reported literature has been carried out with mineral-based lubricants (Gupta and Agarwal 2021; Mujtaba et al. 2020; Ramteke and Chelladurai 2020). The prolonged use of these lubricants significantly contributes to smoke, oxides of nitrogen (NO_*x*_), and hydrocarbon emissions (Tung and McMillan 2004; Mobarak et al. 2014). Apart from being non-recyclable, the biodegradability factor of these lubricants considerably affects the environment during their disposal. Moreover, since these lubricants are derived from non-renewable sources they are prone to cost escalation in relation to demand–supply factors.

Considering the above limitations, researchers are carrying out studies on IC engines using biolubricants, primarily since they are eco-friendly and renewable. Biolubricants are more stable on the viscosity front, exhibiting resistance to changes in temperature due to strong intermolecular interactions, thereby providing a durable and continuous lubricant film between the interacting surfaces (Syahrullail et al. 2013). Amriya Tasneem et al. (2022) investigated wear characteristics and performance of a compression ignition (CI) engine using Karanja-derived biodiesel and a biolubricant. They reported superior performance for a 20% biodiesel blend and overall reduced wear debris detection owing to improved lubricity. According to Shahabuddin et al. (2022), the addition of jatropha oil-based biolubricant in 7.5% concentration with commercial mineral lubricant results in reduced wear rate and coefficient of wear as compared to mineral lubricant in the neat form. Further, for the same blend, superior tribological and morphological characteristics are observed. The use of sunflower oil-based lubricant in a diesel engine exhibits a reduced level of engine emissions with adequate antifriction and antiwear characteristics (Jabal et al. 2019). Lubricant derived from microalgal oil also shows positive impact on engine tribology behaviour, as demonstrated by Cheah et al. (2020) in their investigation using microalgal oil-derived biolubricant in an engine. They report better physicochemical and tribological features for a 10% modified microalgal oil blend. According to Dey and Misra (2017), neat palm oil as biolubricant improves fuel combustion and emission characteristics. Kalam et al. (2017) carried out a comparative investigation of lubrication performance in a CI engine operating on olive oil and mineral oil. They found that olive oil exhibits a 63% of lower coefficient of friction along with better thermal stability up to 390 °C as compared to mineral lubricant. Considering the above, vegetable oil appears to be a promising alternative to mineral lubricant as a biolubricant.

The utilisation of neat vegetable oil/biodiesel as a biolubricant has restrictions, e.g. inferior properties at low temperature coupled with poor oxidative stability in use (Owuna et al. 2020). To overcome these difficulties, the synthesis of biolubricants from vegetable oils can be carried out using processes such as transesterification, epoxidation, and oxirane ring opening reactions (Parente et al. 2021). A study on the use of a biofuel/biolubricant derived from rapeseed oil with copper oxide as nanoadditive on a variable compression ignition engine by Arumugam et al. (2014) indicates that the combined usage of biofuel/biolubricant with a nanoadditive exhibits lower wear, and reduced soot and ash contents, as compared to biofuel/mineral lubricant combination. In addition, improved performance and emissions are observed for the same combination. The heat transfer capability of nanoparticles greatly depends upon their size and concentration (Dinesha et al. 2021). Nano-sized metallic particles have a tendency to accelerate heat transfer when blended with a biolubricant (Atulkar et al. 2021). Ahmad et al. (2022) carried out tribological assessment of biolubricant derived from castor oil with an additive of iron oxide nanoparticles. The study shows that the coefficient of friction and wear are both reduced as compared to neat castor seed oil biolubricant. These findings suggest that the use of metallic nanoparticles in biolubricants enhances the tribological characteristics, leading to improved performance in engines.

Although several performance and emission studies have been carried out on a CI engine using biolubricant with metallic nanoadditives, the study of aluminium-based nanoadditives used with a biolubricant is scant. Further, to understand better the potential performance and emission characteristics of biodiesel with aluminium-based nanoadditives, a thorough literature review was carried out on the suitability of aluminium nanoparticles as a lubrication additive (Luo et al. 2014; Mohan et al. 2014; Sabarinath et al. 2019). The properties like oxidation will be improved when the vegetable oil is subjected to a chemical process known as epoxidation which is the primary requirement of a lubricating oil. Nanoparticles which act as good lubricating agents and a carrier of heat will improve the lubricating properties of the base oil. The combined effect of nanoparticle-added biolubrication and B20 biodiesel fuel combustion may improve engine performance with lower tailpipe emissions. In the present study, the suitability is assessed of using alumina nanoparticles with a biolubricant in a CI engine. The objective is to improve understanding of the effect of gingelly oil-based green fuel and green lubricant with metallic nanoparticles on emission concentrations of HC, CO, and NO_*x*_ and on smoke opacity in a compression ignition engine at varying load conditions.

## Materials and method

### Biodiesel and characterisation

The biodiesel used in the current study is made from gingelly oil and prepared at Mechanical Laboratory, MIT, Manipal, India. The benefit of gingelly oil over other widely available plant-based oils lies in its considerable resistance to deteriorative variations due to the endogenous antioxidants present in it (Menkiti et al. 2015). Gingelly oil comprises 41.5–47.9% linoleic acid and 35.9–42.3% oleic acid under the category of unsaturated fatty acids. It also consists of 7.9–12% palmitic acid and 4.8–6.1% stearic acid, which are saturated fatty acids (Wacal et al. 2019). Since B20 blend has a proven record of superior performance, it is used in this study. The physiochemical properties are assessed according to the relevant American Society for Testing and Materials (ASTM) standards and given in Table 1.

**Table 1:** Physiochemical properties of gingelly oil and its biodiesel blends

Property	Raw gingelly oil	B100	B20	Standard
Flash point (°C)	245	170	88	ASTM D92
Fire point (°C)	275	174	94	ASTM D92
Density (kg/m^3^)	892	882	858	ASTM D4052
Kinematic viscosity (cSt)	26	4.2	3.72	ASTM D 445–03
Lower calorific value (MJ/kg)	39.1	40.2	42.6	ASTM D 240–02

### Biolubricant preparation and characterisation

The biolubricant used here is prepared from gingelly oil via transesterification. It is then subjected to epoxidation to maintain stability at higher operating temperature. Further, alumina nanoparticles are added to the B100 sample in concentrations of 0.5, 1.0, and 1.5% w/w.

#### Epoxidation of gingelly oil ester

Preparing epoxide in a carbon chain using a process is known as epoxidation. The presence of unsaturation in the oils leads to weak oxidative properties and low-temperature properties. The aim of epoxidation is to convert the unsaturation present in the oil to an epoxy ring so as to strengthen these properties.

#### Reaction mechanism

The in situ epoxidation of vegetable oil with carboxylic acid, exposed to a suitable catalyst, to form peracetic acid, is an acid-catalysed reaction, as follows:1

Here, R is the methyl ester in the reaction. It can be seen from the above reaction that RCOOOH is highly unstable and readily losses oxygen atom to form epoxy in place of the double bond or unsaturation prevailing in the oil, as shown in the following reaction:2

In this process, gingelly oil methyl ester, acetic acid, and hydrogen peroxide are used in the molar ratio of 1:0.5:1.5, respectively. Initially, gingelly oil methyl ester and acetic acid are mixed in a conical flask and heated in a magnetic stirrer at 1000 rpm until the reaction temperature of 80 °C is reached. Thereafter, hydrogen peroxide is gradually added over a span of 3 h. Stirring is continued for another 4 h. The solution is then permitted to cool to room temperature. Subsequently, oil is washed with water in a conical glass separator until the impurities are removed and heated to ensure that the entire moisture content in the oil is evaporated.

#### Addition of nanoparticles to biolubricants

The literature review suggests that the use of metallic nanoparticles in biolubricant enhances the performance of IC engines. Considering this, alumina nanoparticles procured from a standard supplier were used in the present investigation. The average sizes of the particles used are 30–50 nm with a purity of 99%. The nanoparticles are added in proportions of 0.5%, 1%, and 1.5% w/w to the biolubricant. The dispersion of nanoparticles in base oil uniformly is ensured using an ultrasonicator. The physiochemical properties of the prepared biolubricants are assessed following ASTM standards (see Table 2). The experimentation is conducted according to the below matrix in Table 3.

**Table 2:** Physiochemical properties of mineral lubricant and epoxidised gingelly methyl ester

Property	Mineral lubricant	Epoxidised gingelly methyl ester
Density (kg/m^3^)	868	878
Kinematic viscosity (cSt)	15.4	4.2
Flash point (°C)	233	183
Fire point (°C)	--	191

**Table 3:** Experimentation matrix

Fuel	Lubricant					
B20	Mineral lubricant (ML)	B100 ester (B100)	B100 ester epoxidised (B100E)	B100 ester epoxidised with 0.5% w/w Al_2_O_3_ (B100E0.5)	B100 ester epoxidised with 1.0% w/w Al_2_O_3_ (B100E1.0)	B100 ester epoxidised with 1.5% w/w Al_2_O_3_ (B100E1.5)

### Experimentation

**Fig. 1:**
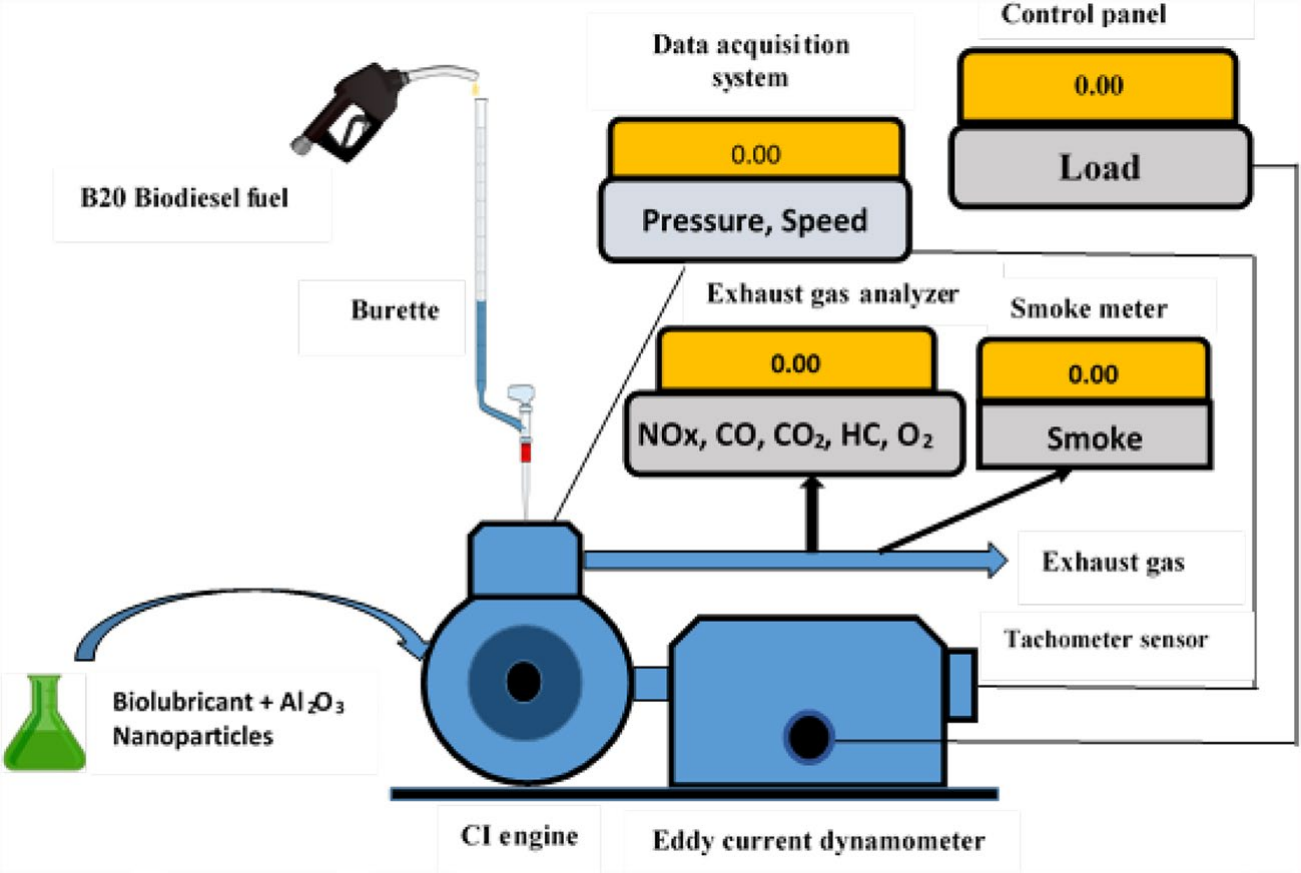
Engine setup

The engine tests are carried out on a 0.66-L capacity single cylinder, naturally aspirated, direct injection CI engine having a compression ratio of 17.5:1 at a rated speed of 1500 rpm. Engine performance is assessed using brake thermal efficiency (BTE) and brake-specific fuel consumption (BSFC). The engine is loaded using an eddy current dynamometer. A water-cooled piezoelectric pressure transducer installed in the engine cylinder is used to acquire cylinder pressure data. The sensor is flush mounted and it can measure the pressure trace in the cylinder with 1° crank angle resolution. The engine performance analysis software “Enginesoft” is used for data acquisition and for carrying out combustion analysis. A schematic diagram of the engine setup is presented in Fig. 1 with corresponding specifications listed in Table 4.

**Table 4:** Specifications of experimental setup

Parameter	Value
Compression ratio	17.5:1
Piston movement (mm)	110
Cylinder diameter (mm)	87.5
Peak brake power (kW)	5.2
Standard injection pressure (bar)	180
Piston displacement (mm)	110
Stroke volume (cm^3^)	661
Rated engine speed (rpm)	1500
Cooling method	Water cooled
Loading method	Eddy current dynamometer
Load sensor	Piezoelectric

Testing is carried out using gingelly B20 biodiesel as a fuel, and gingelly methyl ester (B100), and epoxidised gingelly methyl ester (B100E) and epoxidised gingelly methyl ester (B100E) mixed with 0.5%, 1.0%, and 1.5% w/w alumina (Al_2_O_3_) nanoparticles, as the lubricant combinations. The percentage of nanoparticle used with biolubricant in various studies is less than 2% and reported improved lubrication function (Luo et al. 2014; Mohan et al. 2014; Sabarinath et al. 2019). Hence, in the present study, alumina nanoparticle concentration is limited to 1.5% w/w to avoid agglomeration at higher percentage composition. The engine results obtained are compared with the baseline mineral lubricant operation.

Concentrations of emissions such as NO_*x*_, CO, and HC and smoke opacity are recorded using an AVL exhaust gas analyser and an AVL smoke meter. Table 5 depicts the range and resolution of the measuring devices used in experimentation.

**Table 5:** Instrument range and resolution

Parameter	Instrument range	Measuring resolution
Pressure (bar)	0.344–75	0.0069
*X*_HC_ (ppm)	0–20,000	10
*X*_CO2_ (vol)	0–20%	0.1%
*X*_CO_ (vol)	0–10%	0.01%
*X*_NO_x (ppm)	0–5000	1
Smoke opacity	0–100	0.01

### Engine combustion heat liberation rate

The instantaneous heat liberation rate during combustion is computed as follows (Heywood 2018):

3 $$\frac{{\mathrm{dQ}}_{\mathrm N}}{d\theta}=\frac{\mathrm\gamma}{\mathrm\gamma-1}\mathrm p\frac{\mathrm{dV}}{d\theta}+\frac1{\mathrm\gamma-1}\mathrm V\frac{\mathrm{dp}}{d\theta}$$

where *V* denotes the instantaneous cylinder volume (m^3^), *p* the cylinder gas pressure (bar), *dQ*_N_/*dθ* the instantaneous heat liberation rate per degree crank angle (J/°), *θ* the crank angle (°), and *γ* the ratio of specific heat at constant pressure to specific heat at constant volume, whereas *dV*/*dθ* denotes the instantaneous variation in the cylinder volume per crank angle in degrees. Note also that *dp*/*dθ* denotes the instantaneous variation in the cylinder pressure per crank angle in degrees.

### Uncertainty analysis

Uncertainty analysis is important in assessing the correctness of recorded values in an experimental study. It facilitates assessment of the numerical values of physical variables and how the same are influenced by instrumentation errors. For the current study, the uncertainty of a dependent variable is assessed considering the errors associated with measuring independent parameters like engine load, engine speed, and fuel flow rate, as follows:

4 $${\mathrm W}_{\mathrm R}=\left(\left[\frac{\partial\mathrm R}{{\partial\mathrm x}_1}{\mathrm w}_1\right]^2+\left[\frac{\partial\mathrm R}{{\partial\mathrm x}_2}{\mathrm w}_2\right]^2+\dots+\left[\frac{\partial\mathrm R}{{\partial\mathrm x}_{\mathrm n}}{\mathrm w}_{\mathrm n}\right]^2\right)^{1/2}$$

The uncertainty values assessed for several parameters are given in Table 6. The total uncertainty of the study is 3.75%.

**Table 6:** Uncertainty analysis of recorded parameters for the present investigation

Measured parameter	Percentage uncertainty
Speed	0.51
Load	0.65
Fuel flow rate	0.8
Brake-specific fuel consumption	1.4
*X*_CO_	1.4
*X*_HC_	2.1
*X*_NOx_	2.0
Smoke opacity	0.65

## Results and discussion

Emissions and performance tests are conducted according to the experimentation matrix in Table 3 for 25%, 50%, 75%, and full-load conditions. Engine combustion and performance characteristics such as pressure-crank angle variation, heat liberation rate, and brake thermal efficiency are assessed. Also, *X*_NOx_, *X*_HC_, *X*_CO_, *X*_CO2_, and smoke opacity are recorded. The results are compared with baseline operation for a B20 biodiesel fuel and mineral lubricant combination.

### Cylinder pressure and heat liberation rate

**Fig. 2:**
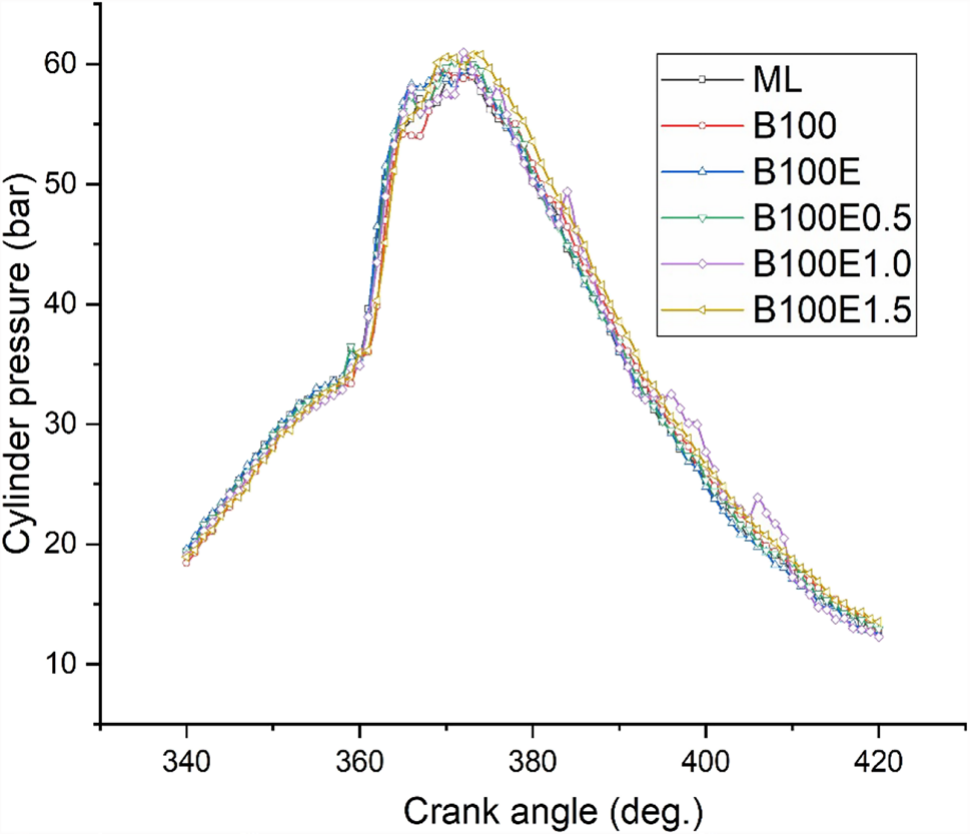
Variation of cylinder pressure for various lubricant combinations at full load

**Fig. 3:**
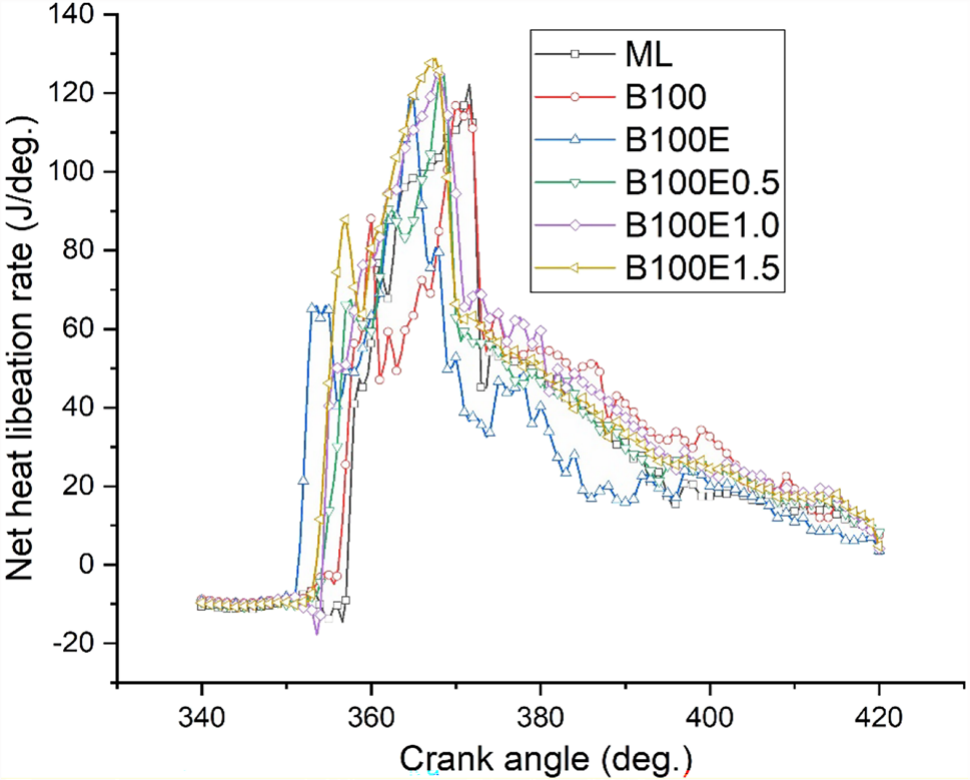
Variation of peak net heat liberation rate for several lubricant combinations at full load

One of the main parameters affecting combustion performance is cylinder peak pressure, which influences brake thermal efficiency and emissions. A higher cetane number coupled with a higher oxygen content of biodiesel promote lower delay period leading to higher heat liberation. The epoxidation of the gingelly oil methyl ester converts carbon to carbon double-bond molecules to highly reactive single-bond molecules with higher oxygen content and leads to better combustion. The piston movement facilitates deposition of metallic nanoparticle-added biolubricant on the cylinder wall, which further enhances combustion due to better heat transfer. This may be possibly attributed to the nanoparticles facilitating heat transfer between the fuel droplets and air (Dinesha et al. 2021). Figures 2 and 3 show the variation of cylinder pressure and heat liberation rate for different lubricants tested. Biodiesel-mineral lubricant operation has a lower cylinder peak pressure and heat liberation rate relative to biodiesel-biolubricant combinations. In addition, biodiesel-biolubricant combinations with Al_2_O_3_ have correspondingly higher values relative to biodiesel-neat biolubricant (B100 and B100E) combinations.

### Brake thermal efficiency

**Fig. 4:**
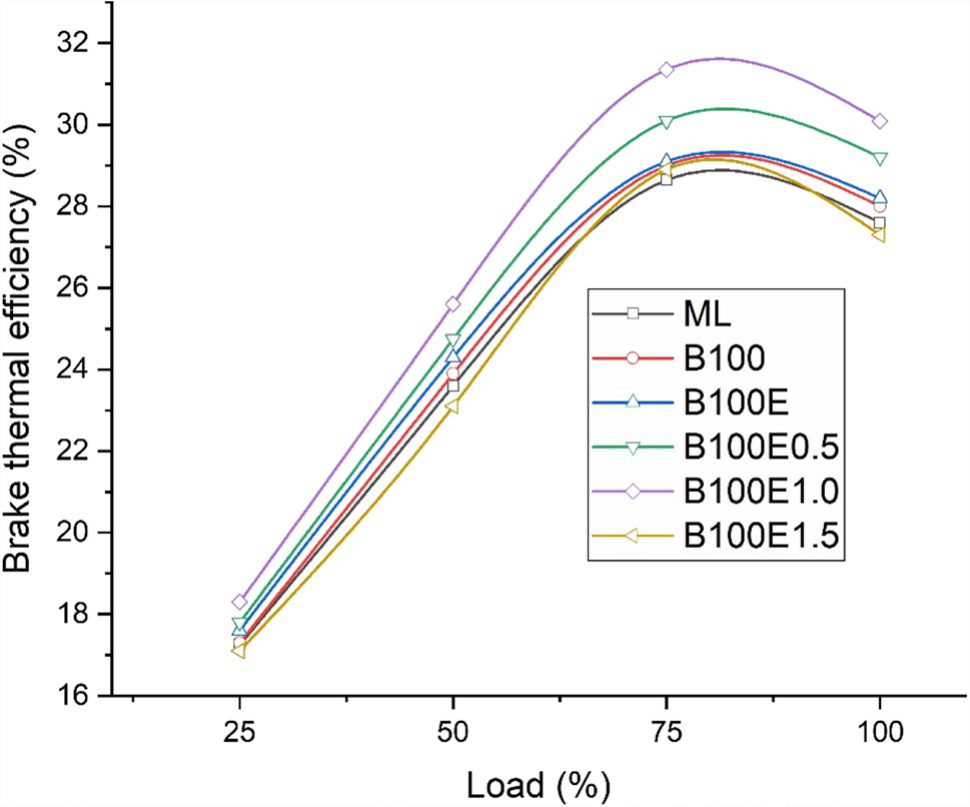
Variation of BTE for several lubricant combinations

In general, BTE increases as the engine load increases, and is observed to be a maximum at a 75–80% loading condition (Mathur and Sharma 2014). Figure 4 depicts the variation of BTE for the lubricants used in the study. Here, the B20 biodiesel-biolubricant combination exhibits superior performance relative to B20 biodiesel-mineral lubricant operation. This may be attributed to the improved lubricity and antifrictional property of the biolubricant. Due to improved viscosity of epoxidised gingelly ester, B100E exhibits better performance compared to B100. Further, the addition of nanoparticles in concentrations 0.5 (B100E0.5), 1.0 (B100E1.0), and 1.5% w/w (B100E1.5) enhances the performance relative to B100 and B100E. The use of nanoparticles results in reduced wear leading to lower frictional losses (Mohan et al. 2014), thereby improving the brake output. A similar study by Yılmaz (2019) reported 15% higher power output for nanolubricants as compared to mineral lubricant operation in IC engines. Amongst B100E0.5, B100E1.0, and B100E1.5, the BTE for B100E1.0 is 31.4%, which is relatively 4.0% and 7.8% higher than the corresponding values for B100E0.5 and B100E1.5, respectively. The rise in BTE for B100E1.0 as compared B100E0.5 may be attributed to the superior antifrictional properties. The increase of nanoparticles from 1 to 1.5% leads to agglomeration of the nanoparticles which intern increases friction, resulting in a lower BTE for B100E1.5.

### NO_x_ concentration in exhaust gas

**Fig. 5:**
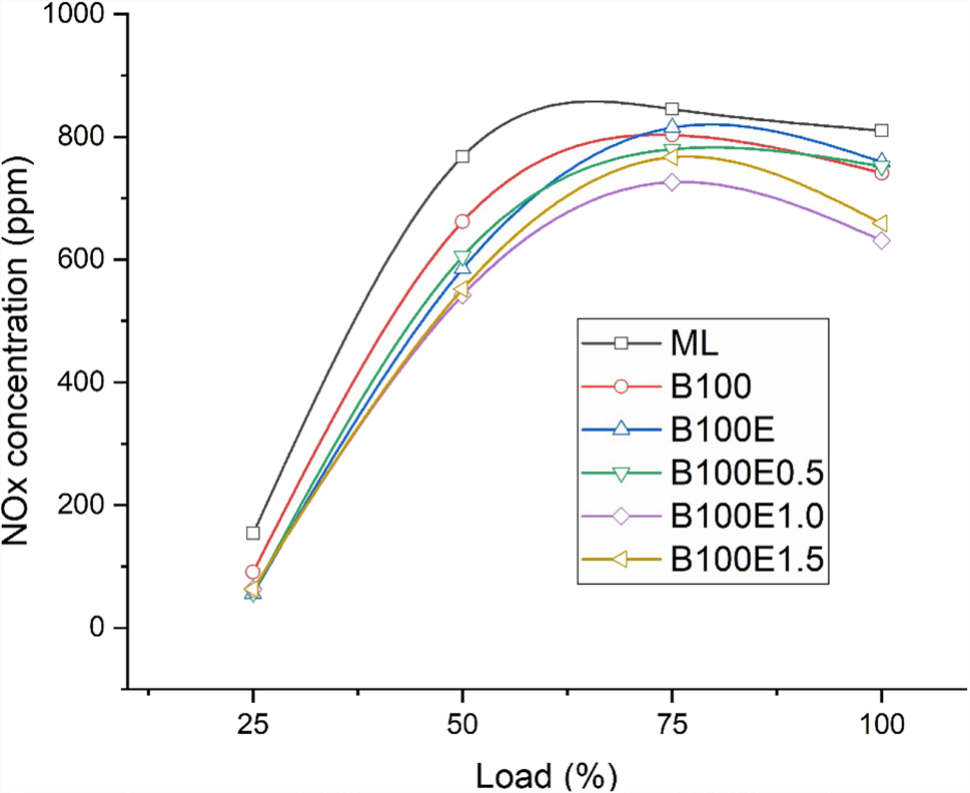
Variation of *X*_NOx_ for several lubricant combinations

The concentration of NO_*x*_ in the exhaust gas increases with load, primarily due to the higher temperature of combustion. The mechanism of NO_*x*_ formation is mainly due to the availability of oxygen, the burnt gas temperature, and reaction residence time (Dinesha and Mohanan 2015). Figure 5 illustrates for the lubricants used in the study the variation of NO_*x*_ emissions. Relative to mineral oil, the use of biolubricants results in lower NO_*x*_ emissions due to the tendency of fatty acids in the biolubricants to unite with the bare metallic contact surfaces, forming a metallic soap. This metallic soap thus formed facilitates more complete combustion due to improved lubricating fluid properties resulting from the adsorption layers (Jabal et al. 2019). Further, the higher heat capacity of the biolubricant facilitates reduction of the reaction temperature, thereby reducing NO_*x*_ formation. The *X*_NOx_ reduction is further improved with the addition of nanoparticles due to effective heat transfer from the burnt gas (Dinesha et al. 2021). The NO_*x*_ concentrations are lower for B100E0.5, B100E1.0, and B100E1.5 by 7.2%, 22%, and 18.6%, respectively, relative to ML. The increase of nanoparticles beyond 1% possibly affects the metallic soap layer formation, thereby influencing combustion and NO_*x*_ formation. Note that a similar study conducted by Atulkar et al. (2021) found a lower NO_*x*_ concentration for nanolubricant in IC engines.

### CO concentration in exhaust gas

**Fig. 6:**
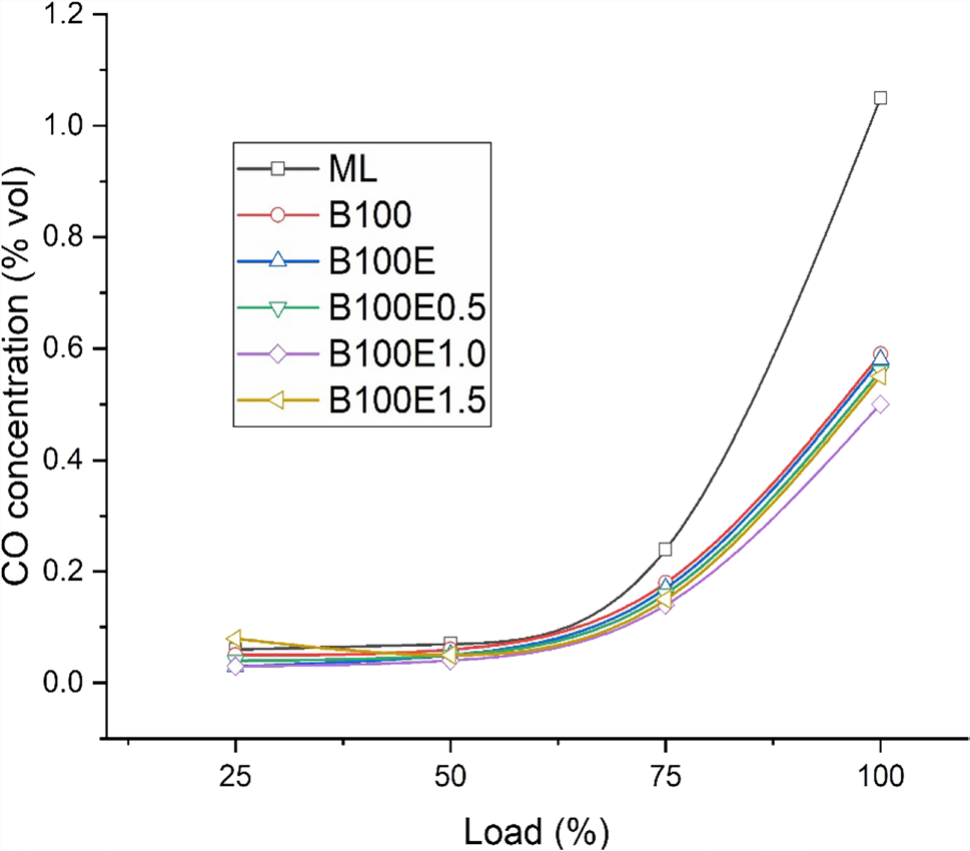
Variation with load of *X*_CO_ for several lubricant combinations

Carbon monoxide formation mainly depends upon the availability of oxygen, combustion duration, and temperature. In general, CO formation increases with load and is observed to reach a maximum at full-load condition due fuel-rich mixture combustion. Equivalence ratio plays an important role during combustion. Biodiesel fuel combustion results in lower CO formation due to the availability of fuel-borne oxygen. Figure 6 depicts the variation with load of *X*_CO_ for different lubricant combinations. Maximum *X*_CO_ is observed for mineral lubricant followed by other biolubricant combinations used in this study. Compared to B100, B100E results in lower *X*_CO_ due to the conversion of carbon to carbon double-bond molecules to highly reactive single-bond molecules. Amongst the three samples of biolubricants with nanoparticles, B100E1.0 exhibits lower CO concentration followed by B100E1.5 and B100E0.5. The CO concentrations are relatively lower by 46.7%, 52.4%, and 4.6% for B100E0.5, B100E1.0, and B100E1.5 samples, relative to ML. A similar study conducted by Yılmaz (2019) found 18% lower CO concentration for nanolubricant compared to baseline operation with mineral lubricant in IC engines. Also, Atulkar et al. (2021) observed similar findings in their study. However, the variation of CO concentration amongst the biolubricants with nanoparticles is observed to be marginally lower.

### CO_2_ concentration in exhaust gas

**Fig. 7:**
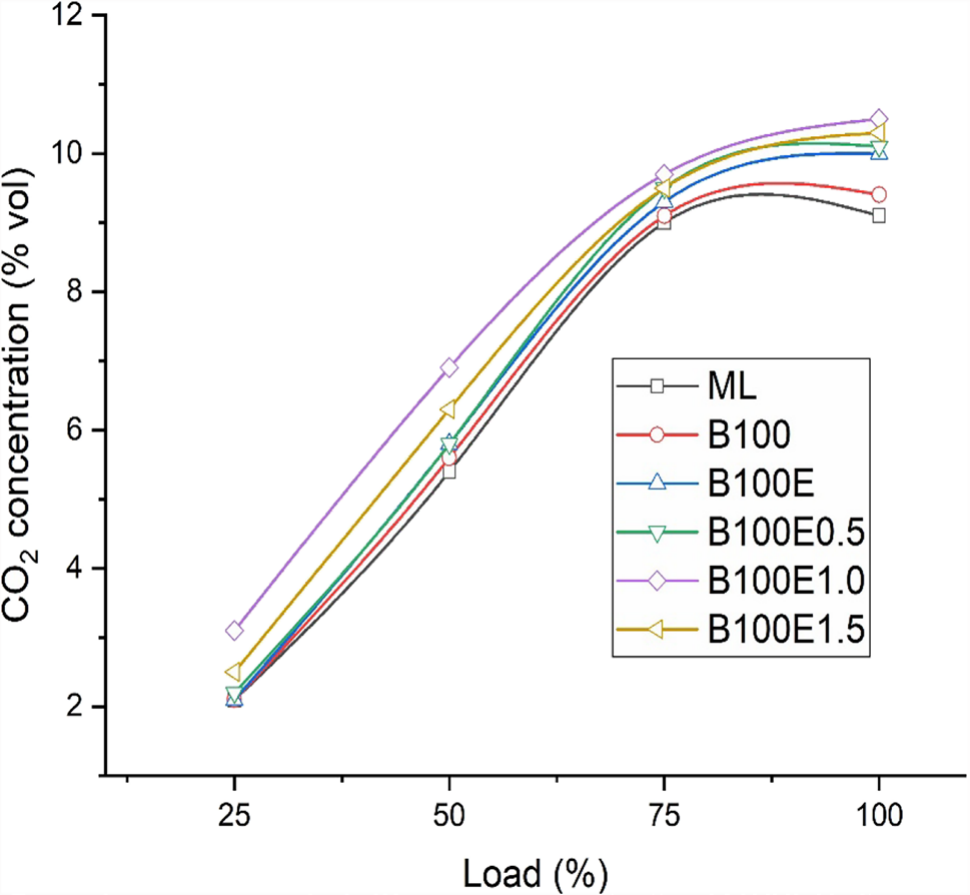
Variation of *X*_CO2_ for several lubricant combinations

CO_2_ is a product of combustion whilst CO is emitted due to incomplete combustion. As the load increases, the concentration of CO_2_ increases due to the higher rate of combustion at elevated temperature. In conformance with the findings related to variation of *X*_CO_ depicted in Fig. 6, similar observations are noted for *X*_CO2_ with an opposite trend. Figure 7 depicts the variation of *X*_CO2_ for various lubricant combinations. Biodiesel-biolubricant combinations result in higher *X*_CO2_ due to inbuilt oxygen being present both in the fuel and lubricant, which leads to more complete combustion. Due to the positive impact of nanoparticle combinations on combustion, higher CO_2_ concentrations are obtained.

### HC concentration in exhaust gas

**Fig. 8:**
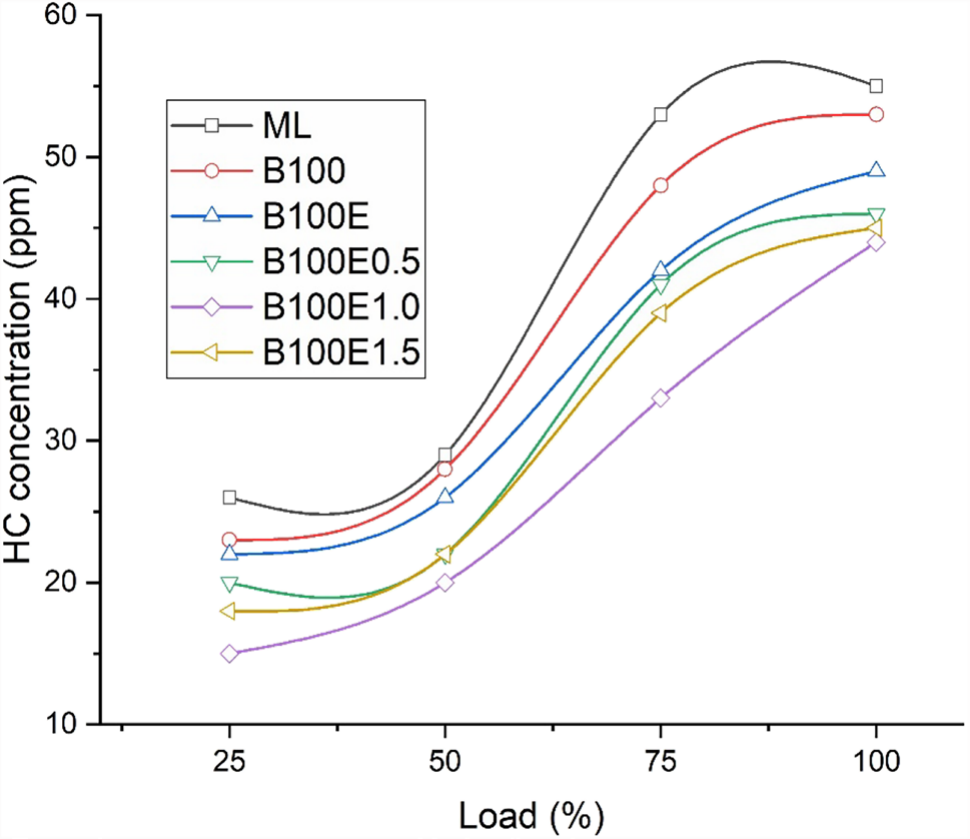
Variation of *X*_HC_ for several lubricant combinations

Hydrocarbon emission is the result of unburnt fuel being discharged with exhaust gases due to incomplete mixture formation resulting from localised concentration of the fuel. In general, HC formation increases with load and is found to be a maximum at full-load conditions due to the supply of a fuel-rich mixture. The variation of concentration of HC for various lubricant combinations is shown in Fig. 8. The biodiesel-biolubricant combination results in a lower concentration of HC emission due to extra oxygen being present in the biolubricant than biodiesel-mineral lubricant combination. Since epoxidation results in a higher concentration of oxygen, the B100E samples exhibit lower HC emissions. The HC concentrations are lower by 3.6%, 10.9%, 16.4%, 20%, and 18.2% for B100, B100E, B100E0.5, B100E1.0, and B100E1.5, respectively, relative to ML. A similar study by Atulkar et al. (2021) reported lower *X*_HC_ for nanolubricants as compared to mineral lubricant operation in IC engines.

### Smoke opacity in exhaust gas

**Fig. 9:**
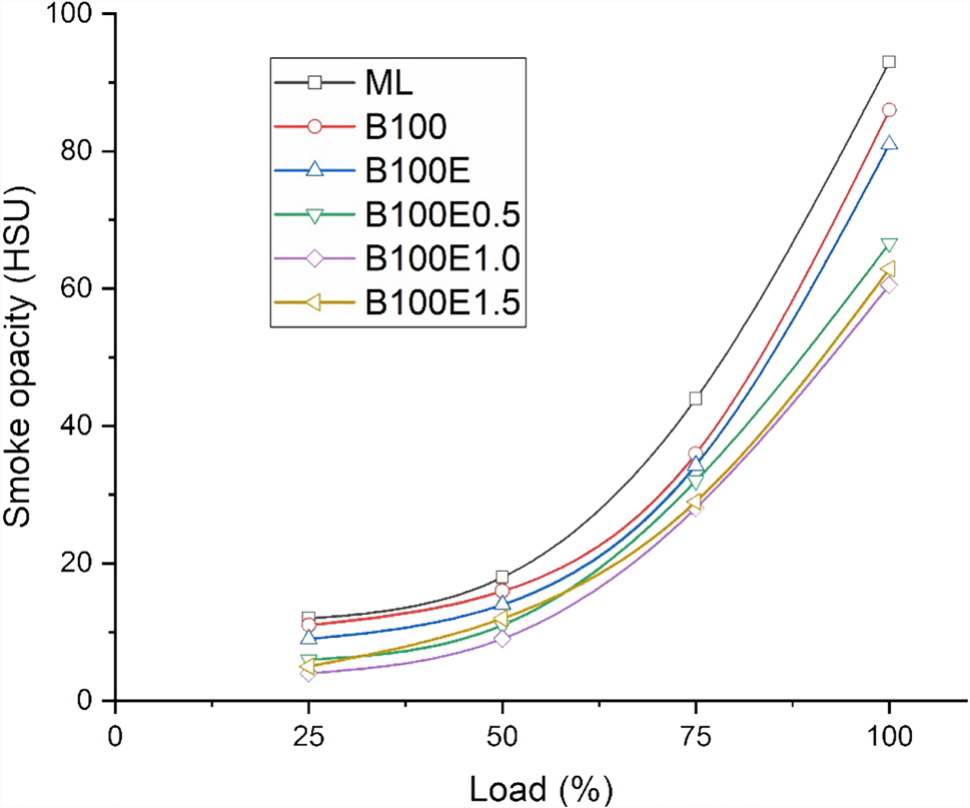
Variation of smoke opacity for several lubricant combinations

Extreme air scarcity leads to smoke formation in a CI engine. The heterogeneous nature of mixture formation leads to uneven concentrations in the combustion chamber of air and fuel. As the air to fuel ratio decreases, the tendency of smoke formation rises. With increasing load, the smoke opacity increases due to combustion of a fuel-rich mixture, as seen in Fig. 9. It is observed that smoke opacity decreases for biodiesel-biolubricant combinations as compared to ML operation. Since the mineral lubricant has a lower affinity for combustion, higher smoke formation occurs. But, the biolubricant has a higher tendency for more complete combustion leading to decreased smoke opacity in line with the behaviour observed for HC emissions. The smoke opacity is found to be lower by 7.53%, 12.9%, 28.39%, 34.95%, and 32.37% for B100, B100E, B100E0.5, B100E1.0, and B100E1.5 samples, respectively, relative to ML. It can be seen that the percentage changes are higher for nanoparticle-added biolubricants as compared to B100 and B100E. This may be attributed to the nanoparticles facilitating heat transfer between the fuel droplets and air (Dinesha et al. 2021).

From the above emission-related observations, it is clear that the use of a green fuel-green lubricant combination leads to a cleaner environment in conformance with the aims of United Nations Sustainable Development Goals SDG7 and SDG13. The green fuel-green lubricants are derived from plant-based sources, which are renewable, and indirectly help to reduce the carbon footprint.

## Conclusions

Experimental investigations are conducted to study the engine performance and tailpipe emissions for a CI engine driven by B20 biodiesel derived from gingelly oil, lubricated with mineral oil, gingelly oil methyl ester, and epoxidised gingelly oil methyl ester with 0.5, 1.0, and 1.5% w/w Al_2_O_3_ nanoparticles. The main results and the conclusions that can be drawn from them follow:

1. The highest BTE is observed for biodiesel-epoxidised gingelly oil methyl ester with 1.0% w/w Al_2_O_3_ combination with a value of 31.4%, which is 8.64% greater than the BTE of biodiesel-mineral lubricant combination.
2. The maximum cylinder pressure and heat liberation rate are also associated with biodiesel-epoxidised gingelly oil methyl ester with 1.0% w/w Al_2_O_3_ combination.
3. Regarding emissions, the opposite trend is observed for *X*_CO_ and *X*_CO2_ with biodiesel-epoxidised gingelly oil methyl ester with 1.0% w/w Al_2_O_3_ combination, resulting in the lowest *X*_CO_, which is 52.4% lower the value for the biodiesel-mineral lubricant combination.
4. The addition of metallic nanoparticles to the biolubricant lead to the formation of a metallic soap leading to more complete combustion, coupled with a higher heat capacity of the biolubricant facilitating reduction of reaction temperature, thereby lowering *X*_NOx_ for B100E1.0.
5. *X*_HC_ and smoke opacity are, respectively, lower by 20% and 34.9% for B100E1.0 as compared to the biodiesel-mineral lubricant combination (ML).

In general, adding Al_2_O_3_ nanoparticles to epoxidised gingelly oil methyl ester not only reduces emissions, but also leads to superior performance. Further, the concentration of the nanoparticles influences engine performance and emissions. The investigation suggests that further studies in this area are merited. The present work addresses SDG 7 (clean energy) and SDG 13 (climate action) through the provision of sustainable energy and facilitating reduced impact on climate change. The study may be extended to explore the possibility of optimising the nanoparticle size added to the biolubricant. Also, tribological feature, SEM, and FTIR analyses of nanoparticle mixed biolubricants need to be better understood, and a lifecycle assessment is required. The present work can be applied to engine-driven power-generating units.

## Data Availability

Data will be made available on request.

## Nomenclature

Al_2_O_3_: Alumina
B100: Gingelly oil methyl ester
B100E: Epoxidised gingelly oil methyl ester
B100E0.5: Epoxidised gingelly oil methyl ester with 0.5% w/w Al_2_O_3_ nanoparticles
B100E1.0: Epoxidised gingelly oil methyl ester with 1.0% w/w Al_2_O_3_ nanoparticles
B100E1.5: Epoxidised gingelly oil methyl ester with 1.5% w/w Al_2_O_3_ nanoparticles
B20: 20% Gingelly oil methyl ester + 80% diesel
BSFC: Brake-specific fuel consumption (kg/kWh)
BTE: Brake thermal efficiency (%)
*dQ*_N_/*dθ*: Instantaneous heat liberation rate per crank angle (J/°)
*dp*/*dθ*: Instantaneous variation in the cylinder pressure per crank angle (bar/crank angle degree)
*dV*/*dθ*: Instantaneous variation in the cylinder volume per crank angle (m^3^/crank angle degree)
HSU: Hartridge smoke unit
ML: Mineral lubricant
p: Pressure (bar)
ppm: Parts per million
rpm: Revolution per minute
V: Volume (m^3^)
CO: Carbon monoxide
CO_2_: Carbon dioxide
HC: Hydrocarbon
NO_*x*_: Oxides of nitrogen
vol: Volume
*X*: Concentration
Γ: Ratio of specific heats
